# Ant brain metabolomics reveals contrasting purine and amine dynamics in a reversible trematode-induced host manipulation

**DOI:** 10.1098/rsos.261298

**Published:** 2026-10-07

**Authors:** Chen-Hua Li, Cameron Goater, James Wasmuth

**Affiliations:** ^1^ Faculty of Veterinary Medicine, University of Calgary, Calgary, Alberta, Canada; ^2^ Department of Zoology, University of Otago, Dunedin, Otago, New Zealand; ^3^ Department of Biological Sciences, University of Lethbridge, Lethbridge, Canada

**Keywords:** host manipulation, altered host behaviour, parasite transmission, Trematoda, biogenic monoamines, adenosine, *Dicrocoelium dendriticum*, *Formica aserva*

## Abstract

The neurochemical mechanisms underlying parasite-induced host manipulations remain poorly understood. Formicid ants infected with lancet fluke, *Dicrocoelium dendriticum*, attach to vegetation with their mandibles during cool conditions, facilitating ingestion by grazing mammals—the obligate definitive host. Following several hours of attachment, ants detach, return to their nests, then repeat the attach/detach sequence on subsequent days. To investigate the molecular basis of this reversible manipulation, we used targeted and untargeted metabolomics to compare the brain chemistry of infected and uninfected field-collected ants during four sequential phases of manipulation. Targeted analysis of biogenic amines showed that tyramine and serotonin concentrations were 16% and 22% lower, respectively, in infected versus uninfected ants, but only during the detachment phase. Untargeted metabolomics revealed that the purine adenosine was 190% higher in infected ants during attachment. This result implicates the adenosine pathway, a key regulator of insect sleep/wake cycles, feeding inhibition and immune responsiveness, in the induction of the immobile, biting phase. Taurine levels were also reduced in the infected ants, potentially suppressing eusocial behaviours such as nest leaving. These results suggest that *D. dendriticum* uses distinct neurochemical mechanisms to orchestrate contrasting phases of manipulation: adenosine-mediated induction of attachment and biogenic amine-mediated regulation of detachment.

## 1. Introduction

Parasites have the potential to manipulate the appearance, morphology and behaviour of their hosts, to facilitate their own transmission [1–3]. Evidence that this potential is realized comes from empirical studies in which parasite-induced alterations enhance transmission of infective stages between hosts [4–6]. Further evidence comes from the results of studies that report changes in tissue concentrations of proteins and other metabolites in manipulated hosts, and changes in gene expression [7–10]. Studies that involve the latter approaches have led to an increase in our understanding of the mechanisms that underlie host manipulations. An understanding of underlying mechanisms of complex host manipulations is possible when changes in features such as host decision-making, activity, foraging and predator-avoidance can be linked to changes in tissue concentrations of neurochemicals involved in their regulation.

Larvae of the lancet liver fluke *Dicrocoelium dendriticum* induce a temporary and reversible behavioural alteration in infected ants [11–14]. Ants that contain even a single larva within the ventral–anterior region of the sub-oesophageal ganglion (SOG) [15] leave their nests, ascend vegetation, then attach firmly with their mandibles onto flower petals or leaf veins. Infected ants remain affixed to the petal during the cooler hours of the day and night, during which they are presumed to have higher rates of accidental ingestion by grazing mammals that are the obligatory definitive hosts. The next day, when temperatures exceed approximately 18°C [11], the ants detach their mandibles, descend the plant and presumably return to their nests. The results of activity monitoring studies show that infected *Formica aserva* repeat the attachment/detachment sequence for the remainder of the summer foraging period [16]. Uninfected ants do not exhibit this sequence of behaviours. During the period when ants are attached to a plant, they do not feed, engage in anti-predator behaviours, or complete typical circadian activity behaviours. Attached ants are also immobile. Thus, not only does the alteration in behaviour involve reversible phases of contraction of the mandible closure muscles, it must also involve reversible alteration to the detection and/or response to typical cues associated with foraging, locomotion and predator detection. Although multidimensional, temporary and reversible host manipulations have been described in other host/parasite interactions [17], underlying mechanisms are not known.

Arguably the most studied potential molecular modulators associated with host manipulation are the biogenic monoamines, particularly thier concentrations in host tissues. These are neurotransmitters, neuromodulators and neurohormones, whose enhancement or disruption can lead to distinctive, often elaborate phenotypic changes in infected hosts. Variation in biogenic amine tissue concentrations has been shown to be closely associated with variation in the expression of altered behavioural phenotypes in parasitoid-infected insects [18–20], acanthocephalan-infected aquatic crustaceans [21–23] and in fluke-infected killifish and lobsters [24,25]. These studies that span numerous host and parasite combinations indicate that the comparison of biogenic monoamine levels in the tissues of hosts of contrasting infection status, or hosts at different stages of manipulation, can provide a starting point for the detection of the chemical basis of host manipulation.

The biogenic amines serotonin, dopamine, tyramine and octopamine play multiple roles in the behaviour of social insects. Serotonin induced biting behaviour accompanied by gaster flexing and formic acid spraying in the ant *Formica polyctena* [26], and it modulated trail-following and foraging behaviour in the ant *Pheidole dentata* [27]. The injection of dopamine induced open mandible threats and biting behaviour in *F. polyctena* [26], whereas starvation decreased brain dopamine concentrations in *F. japonica* workers [28]. Dopamine levels were positively correlated with locomotion in the ants *F. cinereal, Tetramorium caespitum* and *Myrmica scabrinodis* [29], and task specialization in *Acromyrmex echinatior* [30]. Tyramine was involved in threatening and open-mandible aggressive behaviours in *F. polyctena* [26], colony establishment [31] and was positively correlated with locomotion in three species of ant [29]. Octopamine was associated with nestmate recognition in fire ants, *Solenopsis invicta* [32] and with task specialization in *F. polyctena* [33]. These results indicate that the biogenic amines play key roles in the regulation of multiple ant behaviours, including those such as mandible closing, locomotion and foraging that are manipulated by the lancet fluke in infected formicid ants.

In this study, we used targeted and untargeted metabolomics to survey the brains of *Dicrocoelium*-infected and uninfected *F. aserva* across four sequential phases of manipulation: pre-attached, attached, newly detached and detached. In our targeted approach, we compared the concentrations of serotonin, dopamine, tyramine and octopamine across four sequential phases of manipulation. We tested predictions from our previous transcriptomic study, which identified downregulation of enzymes in serotonin and dopamine synthesis during detachment and upregulation of octopamine receptors in pre-attachment [34]. We also sought to determine whether attachment is associated with a chemical ‘on’ trigger that is subsequently turned ‘off’ at detachment, or whether contrasting mechanisms underlie contrasting phases of the manipulation.

## 2. Material and methods

### 2.1. Field collections and experimental design

The source of infected and uninfected ants in Cypress Hills Park, Alberta, together with methods involving the collection, transportation and maintenance of ants in the laboratory, are the same as those described in Li *et al.* [34]. Ants were collected on 17 and 18 July 2021 from three nests at our ‘Staff Camp’ site that have contained infected ants for at least 16 consecutive years. Ants from these same nests have supported our previous field and laboratory studies on other aspects of the *D. dendriticum*/*F. aserva* interaction [34–36].

The experimental set-up for sampling infected and uninfected ants at each of the four sequential phases of the manipulation (pre-attached, attached, newly detached and detached) is the same as described in Li *et al.* [34]. The four phases are nested within two temperatures (10°C and 25°C) and two exposure lengths (short and long exposure) at each temperature: pre-attached = 10°C short exposure, attached = 10°C long exposure, newly detached = 25°C short exposure and detached = 25°C long exposure. In brief, the pre-attached phase was the period when an infected ant had ascended a plant, was opening and closing its mandibles, but was not yet attached. The attached phase was defined as the iconic immobilization period during which an ant was attached firmly onto a flower petal with its mandibles. The newly detached phase was defined as the period when a previously attached ant had relaxed its mandibles for 20–30 seconds from a petal but had not descended the plant. Finally, the detached phase was the period when a previously attached ant was detached from a flower petal for at least 40 minutes.

All ants were maintained at 10°C for 12–72 hours before the start of the experiment [34]. For each phase, ants were randomly selected from holding containers at 10°C and then placed into individual customized perforated plastic containers [16]. Infected ants kept at 10°C remain attached to their flower petals [34]. A similar number of infected and uninfected ants was sampled from the holding containers each day during the 3-day experiment (table 1). To collect samples of newly detached and detached ants, individual ants were transferred from the 10°C chamber to a 25°C chamber. Newly detached ants were sampled within 20 seconds following the relaxation of the mandibles from the flower, whereas detached ants were those that had relaxed their mandibles but were still on the flower on which they were previously attached. Samples of uninfected ants were collected at the same time as samples of infected ants during each phase of the manipulation.

**Table 1.** Daily sequence of *Dicrocoelium*-infected and -uninfected ants sampled for brain tissue amine concentrations at each phase of the manipulation.

conditions	pre-attached		attached		newly detached		detached	
infection status	infected	control	infected	control	infected	control	infected	control
19 July	–	–	38 (57)	37 (38)	4 (4)	4 (4)	49 (54)	42 (43)
20 July	25 (39)	26 (26)	–	–	36 (44)	35 (40)	–	–
21 July	23 (36)	15 (18)	1 (13)	3 (3)	–	–	–	–
Total	48 (75)	41 (44)	39 (70)	40 (41)	40 (48)	39 (44)	49 (54)	42 (43)

***Note***: Numbers in brackets are the total number of individual containers (each with a single infected or uninfected ant) placed into the growth chambers on each day. The number adjacent is the number of ants sampled for amine concentrations. For example, 57 infected ants were placed into a 10°C growth chamber on 19 July; 38 of these attached to plants and were sampled for tissue analysis.

To collect samples of pre-attached and attached ants, individual ants were transferred from the 10°C growth chamber to the 25°C growth chamber to ensure that they detached from their flowers. Infected ants that did not detach at 25°C within 3 hours were discarded. The detached infected ants and uninfected ants were then returned to the 10°C growth chamber. The pre-attached ants were sampled once they showed signs of biting behaviour (i.e. mandible opening and closing). For the attached phase, ants were left at 10°C for at least 40 minutes and sampled once the infected ants were observed attached to the flower. For each treatment, uninfected control ants were placed under the same conditions as the infected group and were sampled at the same time [34].

### 2.2. Targeted metabolomics—amine extraction and analysis

Methods used for sample preparation and ant brain dissection were adapted from Hojo *et al.* [19]. Individual ants were captured, their heads removed, flash-frozen in liquid nitrogen, and then stored at −80°C. We pooled six heads per tube to enhance the probability of detecting biogenic amines in samples of ant brain tissue. Within two weeks of freezing, ant brains were dissected on ice with cold insect cell culture media (Gibco^TM^ IPL-41 Insect Medium). Intact brains (sub- and supra-oesophageal ganglia) were carefully removed from the head capsule without surrounding muscle tissue. The optic lobes and compound eyes were fully excluded to prevent contamination of the sample with pigment. There were six replicates for both infected and uninfected ants in each of the four phases of manipulation, giving a total of 48 samples.

Biogenic amines were extracted using 50% methanol with 0.227 micromolar 3,4-dihydroxybenzyl amine hydrobromide (Sigma-Aldrich) as the internal standard. The concentration of the internal standard added was based on [19]. Each sample containing six brains was homogenized in 49 µl of 50% methanol with 1 µl of internal standard, then chilled on ice for 30 minutes. The samples were then centrifuged at 4°C at 1400 r.p.m. for 10 minutes. The supernatant was then collected for high-performance liquid chromatography–mass spectrometry (HPLC-MS) analysis.

Methods adapted from Hojo *et al.* [19] were used to assess brain concentrations of biogenic amines. The runs were performed on a Q Exactive™ HF Hybrid Quadrupole-Orbitrap™ Mass Spectrometer (Thermo Fisher Scientific) equipped with a heated electrospray ionization probe (HESI-II) coupled to a Vanquish™ UHPLC System (Thermo Fisher Scientific). Chromatographic separation was achieved on an Allure PFP Propyl column (5 mm, 150 × 2.1 mm, Restek) using a binary solvent system at a flow rate of 300 µl min^–1^. The following solvents were used: solvent A prepared with mass spectrometry grade H_2_O with 0.1% formic acid (%v/v), and solvent B prepared with mass spectrometry grade acetonitrile with 0.1% formic acid (%v/v). The gradient elution was as follows: 0–10 minutes, isocratic gradient 10% B; 10–10.5 minutes 98% B; 10.5–12.5 minutes 98% B; 12.5–13 minutes 10% B; 13–16 minutes 10% B. A sample injection volume of 5 µl was used. The column was kept at 40°C.

The mass spectrometer was run in positive full-scan mode at a resolution of 240 000 scanning from 70 to 1000 *m*/*z*. The source settings were the following: spray voltage, 3000 V; capillary temperature, 200°C; sheath gas, 35 (arbitrary unit); auxiliary gas, 15 (arbitrary unit); spare gas, 2 (arbitrary unit); probe heater temperature, 300°C.

The concentrations of tyramine, serotonin, octopamine and dopamine in each sample were calculated on the basis of the calibration curves of each external standard (Sigma-Aldrich) with 0.227 micromolar internal standard. The reported concentrations for each amine (pmol/brain) were calculated from the measurement of each sample, accounting for the 49 µl solution added for extraction and the pooled six brains per sample.

### 2.3. Untargeted metabolomics—extraction and analysis

We performed a general metabolomics survey on the same ant brain samples as described above at the Calgary Metabolomics Research Facility following protocols described in Rydzak *et al.* [37]. Metabolic analyses were performed on a Q Exactive™ HF Hybrid Quadrupole-Orbitrap™ Mass Spectrometer (Thermo Fisher Scientific) coupled to a Vanquish™ UHPLC System (Thermo Fisher Scientific). Chromatographical separation of metabolites was performed on Syncronis HILIC UHPLC column (2.1 mm × 100 mm × 1.7 µm, Thermo Fisher Scientific) at a flow rate of 600 µl min^–1^ using a binary solvent system: solvent A, 20 mM ammonium formate pH 3.0 in mass spectrometry grade H_2_O and solvent B, mass spectrometry grade acetonitrile with 0.1% formic acid (%v/v). The following gradient was used: 0–2 minutes, 100% B; 2–7 minutes, 100%–80% B; 7–10 minutes, 80%–5% B; 10–12 minutes, 5% B; 12–13 minutes, 5%–100% B; 13–15 minutes, 100% B. Sample injection volume was 2 µl. The mass spectrometer was run in both positive and negative full-scan modes at a resolution of 240 000, scanning from 50 to 750 *m*/*z*. Metabolite data were analysed by the El-MAVEN software package [38,39]. Metabolites were identified by matching observed *m*/*z* signals (±10 ppm) and chromatographic retention times to those observed from commercial metabolite standards (LMSLS**^TM^** Sigma-Aldrich).

### 2.4. Statistical analysis

Generalized linear models in R (v. 4.4.2) were used to assess the effects of infection status (infected and control), temperature (10°C and 25°C), exposure length (short and long) and the two-way interactions on the mean tissue concentrations (pmol/brain) of the four biogenic amines in ant brains. Models were fitted using the glm function from the base stats package (v. 4.4.2) with a gamma distribution and log link function, and significance of predictors was evaluated via likelihood ratio tests using the ANOVA function (car package v. 3.1.3). For significant interaction terms, post hoc comparisons were performed using estimated marginal means (emmeans package v. 1.11.2), with pairwise contrasts adjusted for multiple testing. Data visualization was performed using the ggplot2 package (v. 3.5.2). Other brain metabolites identified with the untargeted metabolomics scans were analysed using the same approach, with peak metabolite intensity as the response variable, and the two-way and three-way interactions were included in the model.

## 3. Results

### 3.1. Biogenic monoamines

Each of the four amines was detected in the pooled brain samples of infected and uninfected ants (figure 1; supplementary material, figure S1). The highest amine concentrations were for dopamine (4.54 ± 0.42 pmol/brain, *n* = 48), followed by serotonin (1.05 ± 0.03 pmol/brain, *n* = 48), octopamine (0.77 ± 0.01 pmol/brain, *n* = 48) and tyramine (0.50 ± 0.02 pmol/brain, *n* = 48).

**Figure 1.**
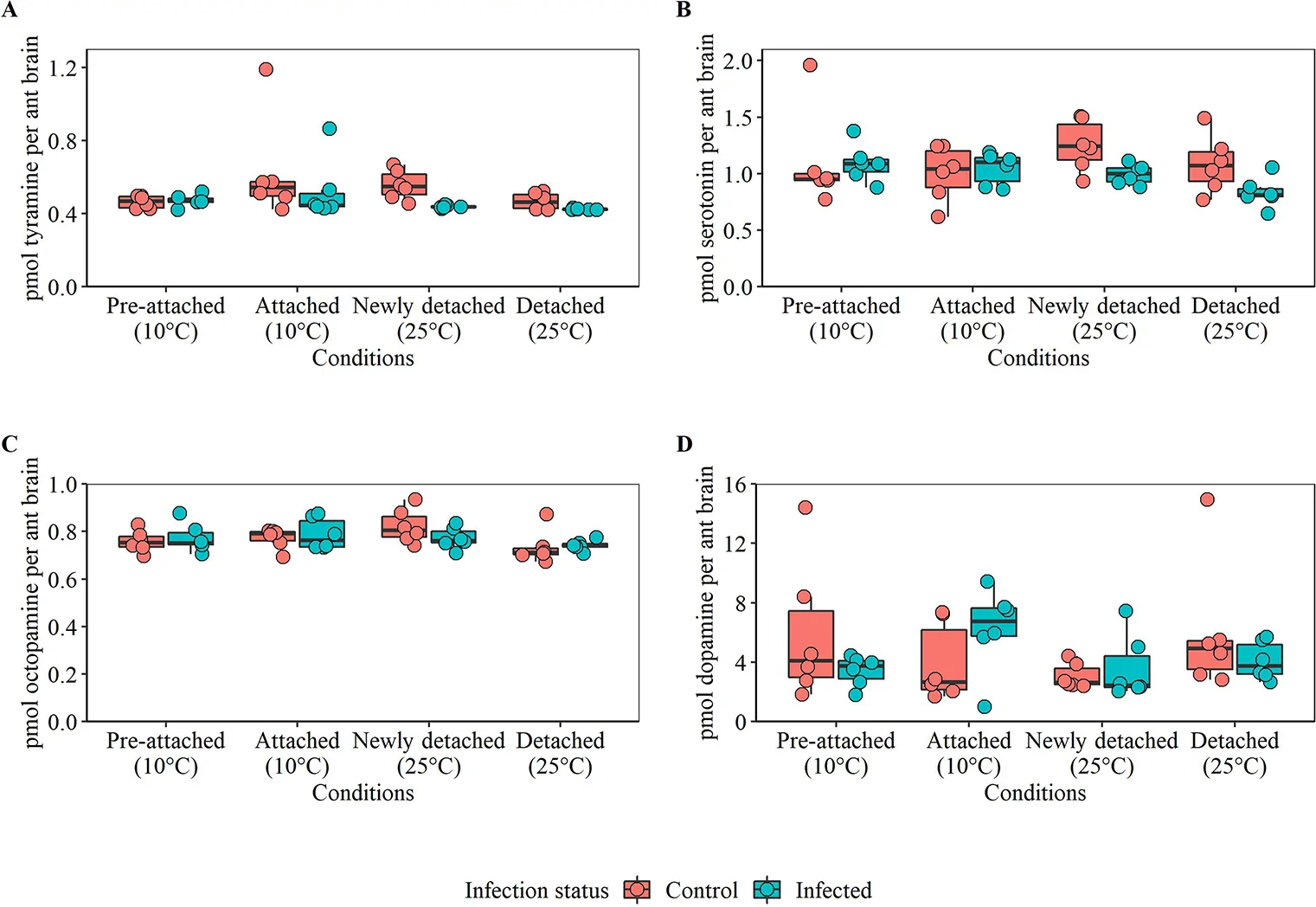
Mean concentrations of the biogenic amines tyramine (A), serotonin (B), octopamine (C) and dopamine (D) in brain tissue of infected and uninfected ants, *F. aserva*, at sequential stages of host manipulation. Data are presented as boxplots in which the horizontal line represents median tissue concentrations and the extensions to the boxes represent the first and third interquartile ranges.

Infected ant brains had 12% lower tyramine concentrations compared with uninfected ants (*χ*² = 4.06, d.f. = 1, *p* = 0.04). In addition, the interaction between temperature and exposure length was significant for tyramine concentration (*χ*² = 6.23, d.f. = 1, *p* = 0.01). Post hoc pairwise comparisons revealed that attached ant brains (both infected and matched uninfected ants) had higher tyramine levels than detached ant brains. This pattern may be influenced by the two brain samples at the attached stage (one infected and one uninfected) that had the highest levels of tyramine concentrations among all of the 48 samples (figure 1). To explore the effects of these two potential outliers on model output, we refitted the model after they were excluded. Infection status and the interaction between temperature and exposure length remained significant, with infection status showing a stronger effect than in the original model (*χ*² = 14.90, d.f. = 1, *p* < 0.001). The interaction between infection and temperature also became significant (*χ*² = 4.90, d.f. = 1, *p* = 0.03). Post hoc tests indicated that tyramine concentrations in infected ant brains at 25°C were 22% and 16% lower than those in uninfected ant brains at both 10°C (*p* < 0.001) and 25°C (*p* = 0.02), respectively.

The interaction between infection and temperature was significant for overall brain concentrations of serotonin (*χ*² = 4.64, d.f. = 1, *p* = 0.03). The results of post hoc pairwise comparisons indicated that serotonin concentrations were 22% lower for infected ants at 25°C compared with control ants at 25°C (*p* = 0.04, figure 2). For octopamine concentrations, the interaction between temperature and exposure length was significant (*χ*² = 5.54, d.f. = 1, *p* = 0.02). The results of post hoc tests showed that newly detached ants (both infected and matched uninfected) had 64% higher brain octopamine concentrations compared with detached ants (*p* = 0.05; supplementary material, figure S1). Ant brain dopamine concentrations did not differ by infection status (*χ*² = 0.19, d.f. = 1, *p* = 0.66), temperature (*χ*² = 0.64, d.f. = 1, *p* = 0.42) or exposure length (*χ*² = 1.77, d.f. = 1, *p* = 0.18).

**Figure 2.**
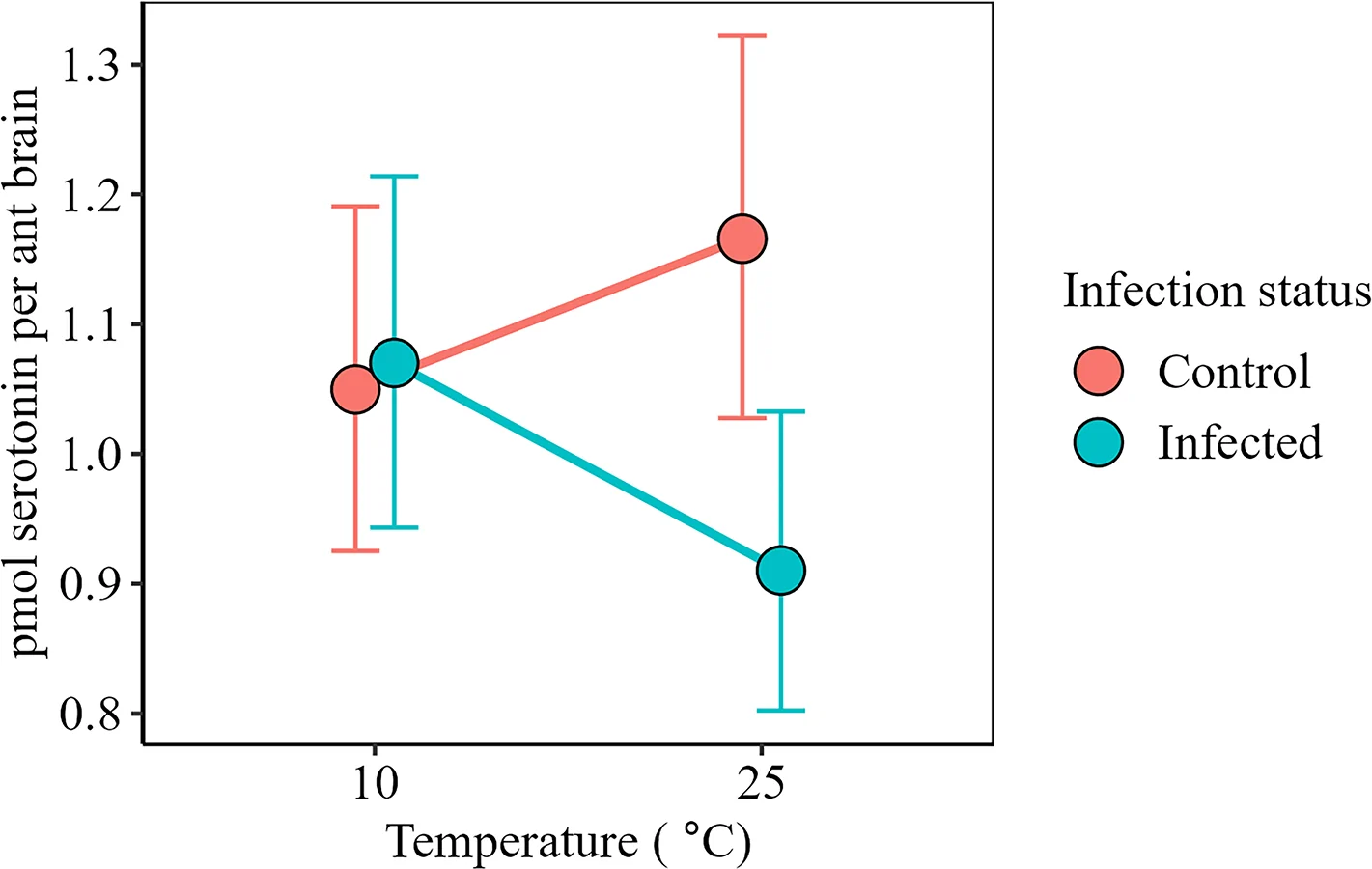
Mean (± 95% CI) serotonin concentrations of *Dicrocoelium*-infected and -uninfected ant brains at two contrasting temperatures.

### 3.2. Untargeted metabolomics

We identified 85 small molecules across negative and positive ionization modes (72 and 48 molecules, respectively, with 35 detected in both; supplementary material, figure S2). Key findings are organized below by infection status and by phase of manipulation.

Several molecules were significantly lower in infected ant brains overall (supplementary material, figure S3), and this pattern was consistent across both ionization modes: taurine (negative: *χ*² = 5.19, d.f. = 1, *p* = 0.023; positive: *χ*² = 6.55, d.f. = 1, *p* = 0.011), inosine (negative: *χ*² = 9.25, d.f. = 1, *p* = 0.002; positive: *χ*² = 6.89, d.f. = 1, *p* = 0.009), AMP (negative: *χ*² = 9.35, d.f. = 1, *p* = 0.002; positive: *χ*² = 10.07, d.f. = 1, *p* = 0.002), IMP (negative: *χ*² = 22.72, d.f. = 1, *p* < 0.001; positive: *χ*² = 13.29, d.f. = 1, *p* < 0.001) and NAD (negative: *χ*² = 13.97, d.f. = 1, *p* < 0.001; positive: *χ*² = 16.39, d.f. = 1, *p* < 0.001). Additional molecules significantly lower in infected ants were detected in a single mode only: allantoin, l-cystathionine, cytidine and alpha-D-glucose-1-phosphate (negative mode) and creatine, l-ornithine and trigonelline (positive mode). D-glucosamine-6-phosphate was the only molecule significantly elevated in infected ant brains overall (*χ*² = 4.91, d.f. = 1, *p* = 0.027, negative mode; supplementary material, figures S2 and S3).

Phase-specific findings were concentrated in the purine pathway. At the pre-attached phase, adenine levels were significantly elevated in infected relative to uninfected ant brains (negative mode: *p* < 0.001; positive mode: *p* = 0.012). At the attached phase, the inhibitory neuromodulator adenosine was 190% higher in infected ant brains compared with uninfected controls (*p* = 0.007, figure 3), representing the most striking phase-specific difference identified by the untargeted approach. At the newly detached phase, infection was associated with significantly lower levels of l-cystathionine (*p* = 0.006), inosine (*p* = 0.018), NAD (*p* = 0.009), hypoxanthine (*p* = 0.002), creatinine (*p* = 0.003) and creatine (*p* < 0.001). This is a broad downregulation consistent with a metabolic reset following the attached phase. At the detached phase, trigonelline was lower in infected ant brains (*p* = 0.020). Additionally, the interaction between infection and temperature was significant for l-cystathionine, inosine and NAD, with infected ants at 25°C showing lower levels than both uninfected ants at 25°C and infected ants at 10°C.

**Figure 3.**
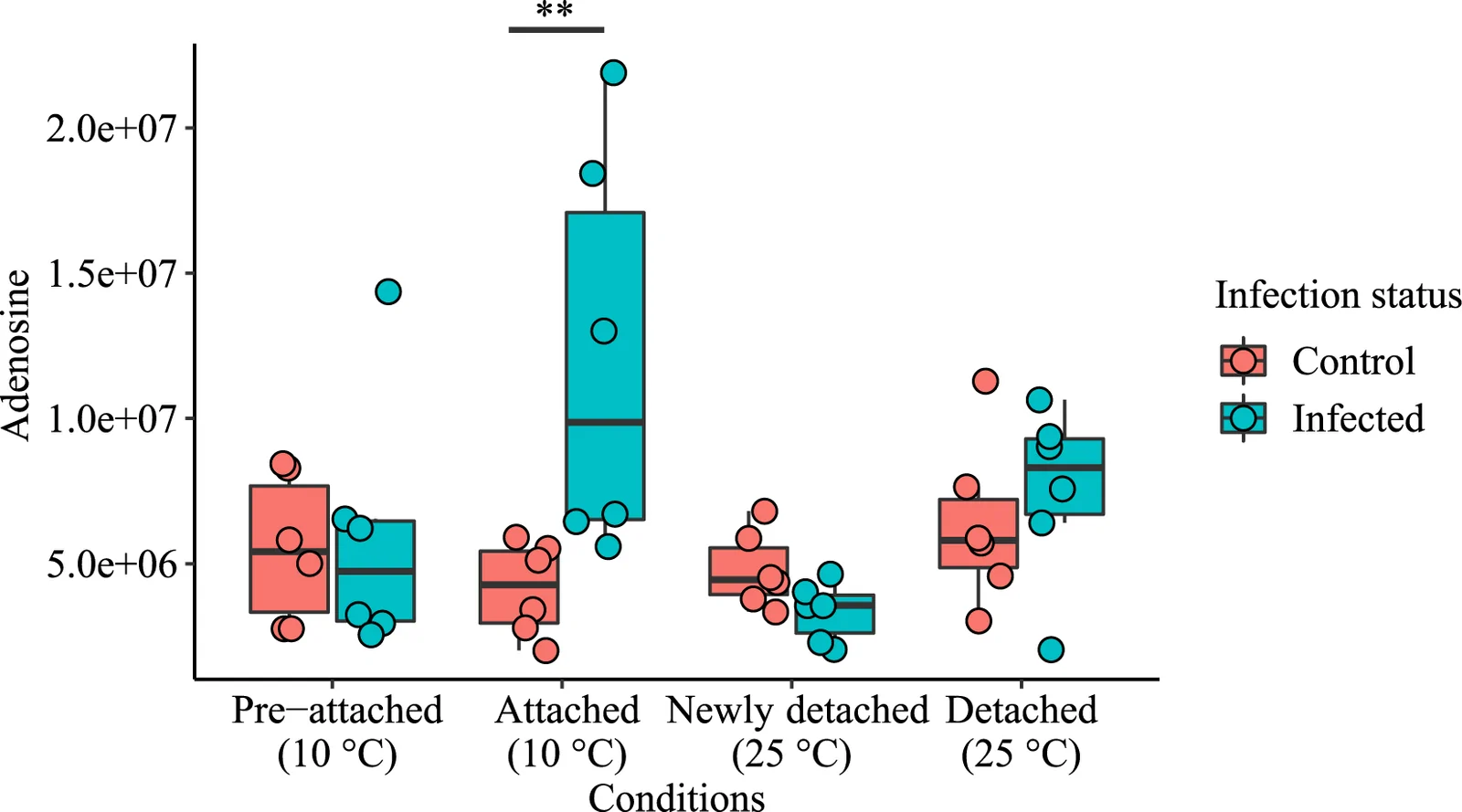
Boxplot of signal intensities (absolute values) of adenosine in *Dicrocoelium*-infected (blue) and -uninfected (red) ant brains. The horizontal line represents median signal intensities, and the extensions to the boxes represent the first and third interquartile ranges. The line and asterisks indicate significant differences between sample groups (**p < 0.01).

## 4. Discussion

We used targeted and untargeted metabolomics to compare the chemical profiles of ant brain tissue in *D. dendriticum*-infected *F. aserva* during different stages of host manipulation. One key finding is that a clear and consistent role for the four biogenic monoamines was not detectable during the iconic ‘biting’ phase of the manipulation. This result implies that the mechanism leading to the contraction of mandibular closure muscles onto flower petals is independent of biogenic monoamine regulation, although we cannot exclude the possibility that infection-associated differences in amine concentrations during this phase were too subtle to detect with these sample sizes. Nonetheless, subtle differences in tissue monoamine concentrations between infected and uninfected ants were observed during other phases of the manipulation, especially during detachment, a result that adds to the growing evidence for a role for amines in host manipulation [18,19,23,40]. A second key finding is that the mechanisms underlying this reversible and multi-faceted manipulation cannot be explained by a single neurochemical trigger that is turned on during one phase of the manipulation, and then reversed during a subsequent phase. In the case of ants infected with larval *D. dendriticum*, contrasting phases of this complex on–off manipulation appear to involve contrasting mechanisms, at least relative to the biogenic monoamines.

Brain tissue concentrations of the amines serotonin and tyramine were reduced in infected compared with uninfected ants, but only during the detachment phase. These results are consistent with our earlier findings that transcripts of the key enzyme associated with serotonin synthesis (tryptophan 5-hydoxylase 1) were downregulated in detached but not attached ants [34]. Both amines play key roles in the regulation of foraging and locomotion in ants. Muscedere *et al.* [27] showed that brain concentrations of serotonin were positively correlated with trail-following and foraging activity in *P. dentata*, and brain tyramine concentrations were positively correlated with locomotion in three ant species [29]. We have observed infected ants ‘lingering’ on their plants following attachment in both laboratory and field settings, and when prodded to detach, they remain on the plant for minutes to hours before reattachment or plant abandonment. By directly or indirectly reducing tissue concentrations of serotonin and tyramine to suppress ant activity following detachment, transmission of infective metacercariae would be facilitated into grazing herbivores, so long as infected ants linger on the plant.

One interpretation for these results is that mechanisms underlying the gradual detachment from a flower petal differ from those involved with the more abrupt and directed behaviours that characterize attachment, such as biting. Herbison *et al.* [1] distinguished punctual host manipulations that occur rapidly from a known start-point from gradual manipulations that occur more slowly and over a longer period. The summiting and biting behaviours that are associated with attachment are consistent with punctual behaviours in Herbison's framework, whereas the lingering and plant abandonment following detachment are more gradual. This distinction may explain why we did not detect a strong association with biogenic amines during the attachment phase, and points towards the existence of a faster-acting amine-independent trigger for the biting behaviour itself.

In the untargeted metabolomics survey, we observed significant downregulation of multiple small molecules related to nucleotide metabolism following detachment, including inosine, hypoxanthine, creatinine, creatine, trigonelline, NAD and l-cystathionine. This broad depletion suggests a rapid metabolic reset, probably associated with the hours-long period of attachment, driven by the purine salvage pathway, which recycles intermediates like inosine and hypoxanthine to regenerate ATP [41,42]. Although our previous transcriptomic data did not show upregulation of the salvage pathway-associated enzymes [34], their activity is probably controlled via post-transcriptional mechanisms, allowing for an immediate response rather than direct synthesis. Notably, ants infected with the fungus *Ophiocordyceps kimflemingiae* also exhibit altered purine metabolism, with reduced AMP and IMP but increased inosine [9], suggesting that this pathway may be a common target for manipulative parasites.

We also observed a significant increase in free adenine in brain tissue during the pre-attached phase, followed by a surge in the inhibitory neuromodulator adenosine during attachment. The accumulation of free adenine probably results from the catabolism of ATP during intense energy expenditure associated with departure from the ant's nest, summitting the plant and then attaching, consistent with our transcriptomic finding of upregulated carbohydrate metabolism in pre-attached ants [34]. There was also an upregulation of adenosine deaminase (ADA) in attached ants [34]. Because ADA catabolizes adenosine, its upregulation during a period of high tissue adenosine levels suggests a compensatory response and may represent a mechanism to prepare the brain for a rapid clearance of adenosine to facilitate return to normal activity upon detachment.

*Dicrocoelium*-induced alteration to the adenosine pathway is a tantalizing candidate for further inquiry. Extracellular adenosine plays a critical role as a signalling molecule for metabolic stress, tissue damage and nutrient regulation in insects, and plays a key role in the orchestration of the insect immune response [43,44]. Overexpression of a single adenosine receptor gene alters sleep–wake cycling in larval *Drosophila*, and adenosine signals in insects are commonly derived from perineural glia, an enveloping membrane that is in direct contact with brain worm in the ventral–anterior-most regions of the sub-oesophageal ganglia [15]. It is conceivable that the large, active brain worm located in the SOG initiates metabolic cascades associated with tissue damage, immune activation, sleep deprivation and general metabolic stress. Each of these, singularly and in combination, could lead to the enhanced levels of adenosine that we observed in attached *F. aserva*, perhaps at the metacercariae/perineural glia interface. We note that this phase of the manipulation is remarkably similar to the sleeping behaviours (i.e. torpor) that are frequently described for some insects, especially hymenopterans, when they overnight on plants, often atop flower petals (review by Lovett *et al.* [45]). Given the linkage between adenosine and sleep/wake cycles in insects, manipulation of the adenosine pathway by brain worm would be a parsimonious and evolutionarily conserved pathway to facilitate metacercariae transmission into grazing mammals.

The sulphur-containing amino acid taurine was also reduced in infected ants. Taurine is found in high concentration in many insect species, including ants, where it is essential for maintaining the central nervous system and the retina [46]. Administration of taurine exerts a positive effect on eusociality—brood care and nest defence—in some species of ant [47]. In crickets (*Nemobius sylvestris*) infected with the nematomorph *Paragordius tricuspidatus*, brain concentration of taurine fluctuated over a 24-hour period, with reduced concentrations coinciding with the period of suicidal water-seeking behaviour [48]. We speculate that reduced taurine in *Dicrocoelium*-infected ants may similarly suppress eusocial responsiveness, promoting abandoning their nest and reducing the probability of departure from the plant during the newly detached phase.

It is very difficult to establish a direct causal link between putative chemical neuromodulators and host manipulation [1]. The problem is especially acute for the multidimensional *Dicrocoelium*-ant manipulation that involves plant summitting, cessation of feeding, immobility and biting. Our comparative metabolomics approach cannot distinguish, for example, whether the surge in tissue adenosine during attachment is caused by infection or by nutrient restriction, inactivity, torpor, long-term muscle contraction, or other factors associated with attachment to a plant. Because infected ant brains naturally contain the brain worm, our metabolomic profiles reflect the combined biochemical environment of the host and parasite. Some elevated metabolites may therefore originate from the parasite itself, although its contribution is expected to be minor given its small size relative to the host brain. The results of this study are important because they identify a set of neurochemical candidates, particularly adenosine, serotonin, tyramine and taurine, that can guide follow-up experimental approaches. Targeted manipulation of these pathways, for example, through exogenous adenosine administration or disruption of receptors, offers a tractable route to establishing causality in this reversible system.

## 5. Conclusion

We present the first metabolomics study of brain tissue changes in *D. dendriticum*-infected ants. Our findings reveal a multi-faceted manipulation strategy in which distinct biochemical pathways underlie contrasting behavioural phases. The adenosine pathway, a key regulator of insect sleep/wake cycles and immune responsiveness, appears to drive the iconic attachment phase, whereas reduced serotonin and tyramine concentrations are associated with the gradual, temperature-dependent detachment and subsequent lingering. Reduced taurine levels may additionally suppress eusocial behaviours, facilitating nest abandonment. Together, these results indicate that *D. dendriticum* uses separate and targeted neurochemical mechanisms to orchestrate its host's complex behavioural sequence, and identify priority targets for experimental investigation of causality.

## Supplementary Material



## Data Availability

All data generated in this study are available in the supplementary material [49].
