# Supplementary material for: Ant brain metabolomics reveals contrasting purine and amine dynamics in a reversible trematode-induced host manipulation
Source: R Soc Open Sci. 2026 Oct 7;13(10):261298. doi: 10.1098/rsos.261298 (PMC13641915; doi:10.1098/rsos.261298)
Supplement: Supplementary file 1 [file rsos.261298.zip › rsos.261298/rsos261298_si_001.docx]

**Supplementary Figure 1.** Mean (± 95% C.I.) transformed octopamine levels per ant brain by temperature, exposure length and infection status.

**Supplementary Figure 2.** Boxplot of the signal intensities (absolute unit, raw or transformed) of the small molecules in *F. aserva*’s brain detected from the negative ionization mode that were influenced by the infection status. The red and blue color indicate the uninfected and *D. dendriticum*-infected ants, respectively. The horizontal line represents median signal intensities and the extensions to the boxes represent the first and third interquartile ranges.

**Supplementary Figure 3.** Boxplot of the signal intensities (absolute unit) of the small molecules in *Formica aserva*’s brain (detected from the positive ionization mode) that were influenced by the infection status. The red and blue color indicate the uninfected and *D. dendriticum*-infected ants, respectively. The horizontal line represents median signal intensities and the extensions to the boxes represent the first and third interquartile ranges.
